# Oral Mucosal Lesions’ propensity as an Outcome Eventuated by Exhaled Carbon Monoxide (CO) Levels and Nicotine Dependency

**DOI:** 10.31557/APJCP.2021.22.9.2781

**Published:** 2021-09

**Authors:** Nidhi Naik, Ridhima Gaunkar, Amita Kenkre Kamat, Vinayak Kamath, Akshatha Gadiyar, Prachi Mungi

**Affiliations:** 1 *Department of Public Health Dentistry, Goa Dental College and Hospital, Goa, India. *; 2 *Goa Medical College and Hospital, Goa, India. *

**Keywords:** Carbon monoxide levels, oral mucosal lesions, Smoked tobacco, Cross-sectional study, nicotine dependence

## Abstract

**Objective:**

This study aims to assess the correlation of exhaled CO and nicotine dependence with the occurrence of oral mucosal lesions while also taking into consideration socio-demographic, clinical and anthropometrical characteristics of participants.

**Methods:**

An observational cross-sectional study was carried out among smokers who visited the tobacco cessation center at Tertiary Care Dental Hospital in Goa, India. An intra-oral soft tissue examination for detecting presence of oral mucosal lesions followed by a questionnaire-based interview for the measurement of exposure, sociodemographic factors, body mass index, cooking habits and nicotine dependence was conducted. The exhaled CO levels were measured with a CO breath analyzer. Statistical analysis was performed using IBM SPSS version 20.0 Descriptive statistics were calculated and multivariable analysis was done to assess the association of different variables with oral mucosal lesions and carbon monoxide levels. p-value ≤ 0.05 was considered as statistically significant.

**Results:**

Of the 173 subjects enrolled in the study, 69.36% were without any lesions while 30.63% were diagnosed with some lesion. In the regression analysis, the variables of physical activity (present vs absent OR: 5.808), exhaled CO levels (OR: 1.098) and nicotine dependence (mild vs moderate OR: 6.518) were significant risk factors influencing the presence of oral mucosal lesions. Usage of both cigarettes and bidis by smokers exhibited highest mean exhaled CO values of 19.67±1.506 ppm. Exhaled CO levels were significantly higher in smokers who were overweight (14.96±9.14 ppm), physically inactive (13.98±8.26 ppm), highly nicotine dependent (20.67±8.30) and used coal for cooking (12.55±8.17).

**Conclusion:**

A robust correlation between exhaled CO levels, nicotine dependence and incidence of oral mucosal lesions was established. The multifactorial tenacity of exhaled CO which is affected by smoked tobacco as well as variables such as physical activity, BMI, cooking habits and type of smoking habit should be noted.

## Introduction

The world is facing ‘The Tobacco Epidemic’ which is proving to be a grave global public health threat. Tobacco kills half of its users accounting to more than 8 million people every year across the globe of which more than 7 million are direct users of smoked or smokeless forms of tobacco while 1.2 million non-smokers lose their life to second hand smoke (World Health Organization, 2020). Today, there are more than 1 billion smokers in the world. (World Health Organization, 2020). Several studies have established the association between tobacco, potentially malignant disorders and oral cancer stating that most oral cancers result from tobacco smoking or using tobacco in other forms (Awan and Patil, 2016; Gupta et al., 2017; Asthana et al., 2019; Mello et al., 2019; Tariq et al., 2020). Thus, tobacco presents to be a crucial contributor towards loss of productivity in developing nations like India ascribable to premature deaths (Chaturvedi et al., 2019; R and Pai, 2018).

In the Indian subcontinent, oral cancer presents to be a major public health problem with it being among the top three types of cancers in the country prevalent in both urban and rural areas (Sharma et al., 2018). With a single puff of smoked tobacco more than 60 well-established carcinogens are inhaled. Critical constituents such as Polycyclic Aromatic Hydrocardons (PAHs), N-nitrosamines, aromatic amines, 1, 3-butadiene, benzene, aldehydes, and ethylene oxide possess high potency amounting to deleterious effects. The toxic effect of tobacco is demonstrated in the form of various oral mucosal lesions, periodontitis, hyperpigmentation, delayed wound healing, leukoedema, salivary glands dilatation, leukoplakia and nicotine stomatitis. Some are simply altered mucosa as a result of the toxic chemicals in tobacco; some are defined as potentially malignant disorders while others might be cases of oral cancer (Gregorczyk-maga et al., 2018).

Incomplete combustion or pyrolysis of tobacco produces a significant amount of carbon monoxide (CO) which occurs due to the thermal decomposition and combustion of various tobacco components such as starch, cellulose, sugars, organic acids, esters, etc. A concentration of 400-500 ppm of CO is delivered when a single cigarette is smoked. A person smoking a pack of cigarettes per day would have an exhaled CO level of approximately 20 ppm. However, in addition to this, the atmosphere too, usually has a concentration of CO less than 0.001% but it may be higher in urban areas due to automobile emissions and industrial production (Dorey et al., 2020). In rural and semi-urban India, there is frequent use of indoor coal-burning heaters and stoves during winter months. Practices of using wood and other indigenous forms of cooking equipment are also not uncommon giving rise to increased CO emissions(Sikary et al., 2017). 

CO acts as a blood poison by forming carboxyhemoglobin (CoHb), a stable complex with hemoglobin, and disrupting the exchange of oxygen in the blood on entering into the blood through the lungs (Djulančić et al., 2013). Other than hypoxic tissue injury, CO toxicity can lead to reactive oxygen species formation, deregulation of ion channels, alteration of cytochrome C oxidase and the cellular respiration as well as mitochondrial function, or endothelial nitric oxide release (Gregorczyk-maga et al., 2018). Exhaled CO level has reportedly been utilized as an effective indicator of smoking in clinics and hospitals. This level can be measured using a portable and non-invasive CO monitor for assessing an individual’s smoking status (Hung et al., 2006).

Although there have been published studies conducted till date reiterating the association of smoked tobacco with oral cancers, there is a scarcity of studies taking into consideration other sources of CO related to local cultures and lifestyle which may have an effect on the readings noted (Gupta et al., 2017; Mello et al., 2019). There is an impending need to fill the lacunae in existing published literature by investigating other sources of CO and their possible correlation with the occurrence of oral mucosal lesions coupled with potential triggering risk factors, which when not taken into purview contribute as confounding variables. The current study bridges this gap by comprehensively determining the association between exhaled CO levels, nicotine dependence and oral mucosal lesions among active as well as active and passive combined cigarette and bidi smokers, while also taking into consideration socio-demographic, clinical, anthropometrical and other characteristics such as co-morbidities, Body Mass Index (BMI), physical activity etc. which might have an association with these lesions.

## Materials and Methods

This study employed an observational cross-sectional study design and was carried out in accordance with the STROBE guidelines for cross-sectional studies (Von Elm et al., 2008). It was conducted at the Tertiary Care Dental Hospital in Goa, India for a period of 2 months from January 2020 to February 2020. The research was conducted following the principles of the Declaration of Helsinki, the necessary permissions for which were obtained from the Institutional Ethics Committee (GDCH/IEC/I-2020-4).

Oral Mucosal Lesions (OMLs) were classified following the WHO criteria which include Leukoplakia, Submucous fibrosis, Erythroplakia, Actinic keratosis, Palatal lesions in reverse smokers, Leukoedema , Lichen planus and Discoid lupus erythematosus (Warnakulasuriya et al., 2007). Smokers were defined as daily or almost daily smokers, who had smoked at least 100 cigarettes in their lifetime and passive smokers were individuals who inhaled second-hand smoke exhaled by smokers. A pilot test was carried out on 30 individuals to check for the flaws and feasibility of the study who were not recruited in the final sample. The G* Power Statistical Software (version 3.1.9.2) was utilized which estimated a sample size of 173 subjects. The study undertook a convenience sampling technique and consecutively included smokers, who visited the tobacco cessation center at Department of Public Health Dentistry during the study period, were older than 18 years of age and willing to participate in the study. Those with presence of asthma; chest deformation causing breathing problems; influenza like illness in the preceding week of the test; Chronic Obstructive Pulmonary Disease; on-going neoplasm disease; lung resection; history of lung cancer and smokeless tobacco users were excluded from the study. All subjects gave a written informed consent indicating their voluntary and anonymous participation in the study.

The study protocol included an intra oral soft tissue examination and a questionnaire-based interview for the measurement of exposure. Presence of OMLs was assessed by two clinicians, who reflected almost perfect inter-rater reliability of Κ= 0.92, under standardized conditions, artificial lighting and using a mouth mirror. In addition, further diagnosis was confirmed by available histo-pathological report. Carious lesions, endodontic lesions and periodontal lesions were excluded from the oral lesions studied. The participants completed a questionnaire that collected demographic data and information addressing issues such as sociodemographic factors, BMI, physical activity, systemic chronic diseases and smoking habits including the frequency and length of smoking history, current smoking status and exposure to passive smoking. Modified Kuppuswamy scale (Wani, 2019) which consists of a composite score including the education and occupation of the Family Head along with income per month of the family was used to measure socio economic status (SES). BMI was calculated by dividing the subject’s weight (in kilograms) by height (in meters squared). BMI limits for underweight (< 18.5), normal (18.5 to 24.9), overweight (≥25 to 29.9), and obesity (≥30) were used for classification of subjects (Gallagher et al., 2000).

Nicotine dependence amongst the habitués was assessed by filling in the modified Fagerstrom test (Ebbert et al., 2006) which is a standard instrument designed to provide an ordinal measure of physical addiction to cigarette smoking. A score of less than 4 points was classified as minimal dependence; score of 4 to 6 as moderate dependence and score of 7 to 10 as high dependence on tobacco. The exhaled CO levels were measured in all participants with a MicroSmokelyzer (Bedfont Scientific Ltd., Kent, United Kingdom), a breath CO monitor intended for multi-patient use. Tests were performed at least 30 min after the last cigarette smoked or exposure to passive smoking in accordance with the recommendations included in the device manual. After automatic calibration, participants were asked to exhale completely, inhale deeply and after holding their breath for 15 seconds exhale slowly and fully into the analyzer. Also, patients were instructed on how to exhale in the mouthpiece of the monitor to ensure that no air escaped from the measuring device while exhaling. All samples were collected in the dental cabinet where smoking is not permitted. The results were immediately displayed on the screen, showing the exhaled CO levels in parts per million (ppm). The percentage of hemoglobin combined with carbon monoxide (CoHb concentration) was predicted by using the Jarvis curve (CoHb = 0.63+0.16 x CO) (Andersson and Moller, 2019).

Data was analyzed using IBM SPSS (Statistical Package for the Social Sciences version 20.0, Chicago). Descriptive statistics were presented in the form of frequencies, percentages, mean and standard deviation. Normality of the data was assessed prior to analysis using Shapiro-Wilk’s test and was found to be normally distributed. Pearson’s chi-square test (χ2-Test) was applied to determine differences of qualitative data. Odds ratio (OR) with 95% confidence interval was used to measure the strength of the association between smoking and oral mucosal lesions and adjusted odds ratio was obtained using multivariate logistic regression. Comparison of mean CO levels was compared between the study groups using independent sample t test. Multiple linear regression was used to assess the influence of study variables on CO levels. p-value ≤ 0.05 was considered as statistically significant.

## Results

A total of 173 participants enrolled in this study of which 171 were males and the mean age was 37 years. On the basis of their social economic status, majority of them belonged to upper lower (49.1%) and lower middle class (32.36%). Of the 173 subjects, 69.36% were without any lesions while 30.63% were diagnosed with some OML. The percentage distribution of various OMLs amongst the smokers is presented in Figure 2. No subjects with lesions and 2 subjects without lesion had a familial history of cancer. 11.3% with lesions had existing co-morbidities (Diabetes and Hypertension). Physical activity in any form was seen in 46.24% of the participants. Of all the participants in whom presence of lesion was noted 67.9% were exposed to both active and passive smoke while 32.1% were active smokers. High nicotine dependence (24.5%) was observed in smokers with OMLs as compared to 6.7% in habitués without lesions. Physical activity (p = 0.013), more no. of units of cigarettes/ bidis smoked (10.13±10.6>6.25±8.4, p= 0.021) as well as lesser time elapsed since the number last smoke (284.15±270.4<1679.94±4499.8 seconds, p= 0.001) and exhaled carbon monoxide levels (15.23±7.5>10.34±7.7, p<0.001) had a statistically significant impact on the presence of lesions in the participants (Table 1).

Of all the predictors in Table 2, the regression analysis computed that the variables of physical activity (present vs absent OR: 5.808), exhaled CO levels (OR: 1.098) and nicotine dependence (mild vs moderate OR: 6.518) were significant risk factors influencing the outcome that is presence of oral mucosal lesions with p values of less than 0.05.

When CO levels were compared among various types of smoked tobacco it was found that those smoking both cigarettes and bidis had highest mean exhaled CO values of 19.67±1.506 ppm followed by those smoking bidis only 17.41±11.041 ppm and cigarettes only 10.89±7.283 ppm. CO levels were significantly higher in overweight (14.96±9.14 ppm), obese (11.23±9.42), physically inactive (13.98±8.26 ppm) and highly nicotine dependent (20.67±8.30) individuals. Smokers cooking using coal also exhibited significantly higher CO levels (12.55±8.17) (Table 3).

The various predictors mentioned in Table 4 could explain 46.6% variation in the outcome that is the CO levels in the exhaled air of the subjects. Of these variables’ physical activity, co-morbidities, BMI, Fagerstrom score, cooking habits (gas stove, coal, combination of gas and kerosine) and type of smoking habit (combination of bidi and cigarette) significantly predicted the outcome. The variable ‘cigarette’ type of habit was strongly correlated to the type ‘bidi’ (84% correlation) and hence got automatically eliminated from the regression model.

**Figure 1:**
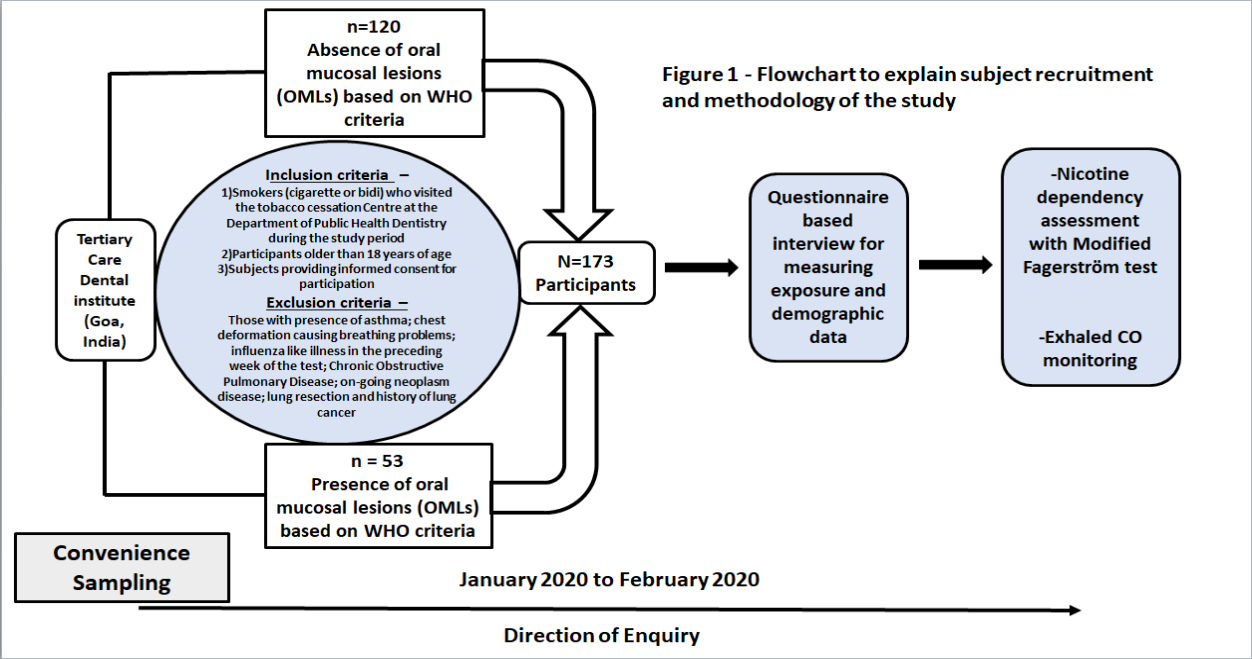
Flowchart to Explain Subject Recruitment and Methodology of the Study

**Table 1:** Sociodemographic, Clinical, Anthropometrical and other Characteristics of Study Subjects with and without Oral Mucosal Lesions (OMLs)

Variable compared	Categories	Without OML (120)	With OML (53)	p value
		No. (%)	No. (%)	
Gender	Males	118 (98.3)	53 (100)	
	Females	2 (1.7)	0	0.34 ^1^
SES	Lower	4 (3.3)	4 (7.5)	
	Upper lower	62 (51.7)	23 (43.4)	0.001*^1^
	Lower middle	45 (37.5)	11 (20.8)	
	Upper middle	9 (7.5)	15 (28.3)	
Physical activity	Present	63 (52.5)	17 (32.1)	
	Absent	57 (47.5)	36 (67.9)	0.01*^1^
Co- morbidities	Present	9 (7.5)	6 (11.3)	
	Absent	111 (92.5)	47 (88.7)	0.41 ^1^
Smoking status	Passive & active	68 (56.7)	36 (67.9)	
	Active	52 (43.3)	17 (32.1)	0.16 ^1^
Smoke tobacco type	Bidi	7 (5.8)	10 (18.9)	
	Cigarette	110 (91.7)	40 (75.5)	0.01*^1^
	Bidi and cigarette	3 (2.5)	3 (5.7)	
Family history of tobacco use	Present	42 (35)	26 (49.1)	0.08 ^1^
	Absent	78 (65)	27 (50.9)	
Family history of cancer	Present	2 (1.7)	0	
	Absent	118 (98.3)	53 (100)	0.08 ^1^
Nicotine Dependence	Low	102 (85)	34 (64.2)	
	Medium	10 (8.3)	6 (11.3)	0.002*^1^
	High	8 (6.7)	13 (24.5)	
				
Age (in years)		37.26 ±13	37.91±11.9	0.76 ^2^
Age of initiation (in years)		24.29±9.9	23.47±5.6	0.49 ^2^
Body mass index		24.93±5.2	26.35±3.6	0.40 ^2^
No of units smoked per day		6.25±8.4	10.13±10.6	0.02* ^2^
Time elapsed since last smoke (in minutes)		1679.94±4499.8	284.15±270.4	0.001* ^2^
Carbon monoxide level		10.34±7.7	15.23±7.5	<0.001* ^2^
CoHb level		2.28±1.2	3.06±1.2	<0.001* ^2^

^1 ^Chi Square Test; ^2^ Independent sample t test; *p<0.05 Statistically Significant; p>0.05 Non-Significant

**Figure 2:**
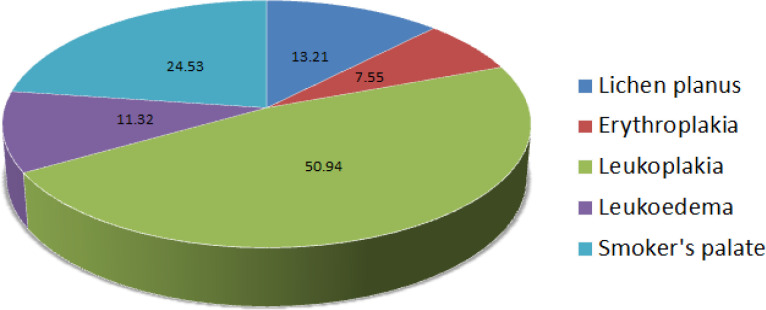
Percentage Distribution of Oral Mucosal Lesions

**Table 2:** Regression Analysis with Independent Variables Predicting the Outcome- ‘presence of lesion"

Variable	Odds ratio ( Confidence interval)
SES: ref-upper middle	
SES (1)-lower	0.51 (0.07 – 3.64)
SES (2)-upper lower	0.20 (0.02 -1.78)
SES (3)- lower middle	1.40 (0.17 – 11.47)
Physical activity: ref - present	
Absent	5.81(2.00 – 16.85)
Type of smoke tobacco used: ref-bidi	
Type of smoke tobacco used (1)- cigarette	0.62 (0.15 – 2.61)
Type of smoke tobacco used (2)-both	1.57 (0.17 – 14.97)
No of units smoked per day	1.13 (0.88 – 1.24)
CO level in exhaled air in ppm	1.10 (1.02 – 1.18)
Nicotine Dependence (Ref – Mild)	
Moderate	6.52 (1.46 – 29.08)
Severe	1.74 (0.37 – 8.05)

**Table 3:** ‘Carbon Monoxide Levels in Exhaled air’

Variable compared	Categories	Mean (S.D)	p value
Gender	Males	11.82±8.0	0.84 (NS)
	Females	13±0	
SES	Lower	8.5±4.04	0.04**
	Upper lower	12.32±6.85	
	Lower middle	10.20±8.24	
	Upper middle	15.08±10.61	
Body Mass index	Underweight	7±3.82	0.002*
	Normal	10.39±5.9	
	Overweight	14.96±9.14	
	Obese	11.23±9.42	
Physical activity	Present	10±7.23	0.001*
	Absent	13.98±8.26	
Co- morbidities	Present	19.60±11.81	<0.001*
	Absent	11.10±7.12	
Mode of cooking	Gas	6.71±2.91	<0.001*
	Other methods	12.55±8.17	
Smoking status	Passive & active	12.06±8.93	0.63 (NS)
	Active	11.51±6.25	
Smoke tobacco type	Bidi	17.41±11.041	<0.001*
	Cigarette	10.89±7.283	
	Bidi and cigarette	19.67±1.506	
Nicotine Dependence	Low	10.32±7.07	<0.001*
	Medium	13.13±7.31	
	High	20.67±8.30	

Independent sample t test; *p<0.05 Statistically Significant, p>0.05 Non-Significant, NS; **Post hoc test – There was statistically significant difference in exhaled CO levels between upper middle and lower middle (p value = .010); upper middle and lower (p value = .026); upper lower and lower (0.012)

**Table 4:** Regression Analysis with Independent Variables Predicting the Outcome- ‘Carbon Monoxide Levels in Exhaled air’

Variable	B (SE)	T value	P value	95 % CI lower limit	95 % CI upper limit
Constant	-1.7	-0.53	0.60 (NS)	-8.03	4.63
SES	2.81	4.04	<0.001*	1.44	4.19
Physical activity	-3.67	-3.35	0.001*	-5.84	-1.51
Co-morbidities	4.78	2.84	0.005*	1.45	8.11
Cooking Habits	-6.76	-3.93	<0.001*	-10.15	-3.36
BMI (kg/m^3^)	0.21	2	0.047*	0	0.41
Nicotine Dependence	1.22	5.7	<0.001*	0.8	1.64
Type of smoke tobacco					
Bidi	2.72	1.52	0.13 (NS)	-0.82	6.25
Both	6.77	2.41	0.02*	1.22	12.31
R ^2^ change (p value)	0.47 (<0.001*)				

*p<0.05 Statistically Significant; p>0.05 Non-Significant, NS

## Discussion

A kaleidoscopic blend of Indian and Portuguese cultures, Goa is India’s most sought-after tourist attraction. As much as urban Goa is known for its pristine beaches, it is also popular among the youth for being the ultimate party destination which implies easy access to all forms of tobacco products, both smoked and smokeless. Several surveys have found higher prevalence of smokeless tobacco use among individuals belonging to rural and urban slums where more than 60% of the population dwells. This is ascribable to the high illiteracy rates, failed outreach of anti-tobacco campaigns, unemployment and laborious jobs. (Central Statistics Office, 2013; Thakur and Paika, 2018; Diendéré et al., 2020) Moreover, frequent power cuts, indigenous cooking appliances and orthodox heaters generating CO remain popular in these households leading to increased environmental CO levels. As the present study setting is the sole government dental institute in Goa, it stands as litmus to the urban-rural composite make of the resident population and beneficiaries attending the same. This representation makes the results extrapolatable and generalizable.

The product of incomplete combustion of carbonaceous material, CO is a non-irritating, tasteless, odourless and colourless gas. It is most commonly found in cigarette smoke, automobile exhaust, improperly ventilated homes utilizing heating units and indigenous cooking appliances like gas stoves, kerosene stoves or wood stoves. Second hand smoke may also contain high levels of CO. In heavy smokers, the oxygen level can be decreased to 15% and they may suffer from CO poisoning if smoking is carried out in enclosed spaces (Lippi et al., 2012). To ensure establishment of a robust association between the prevalence of oral mucosal lesions and increased exhaled CO levels, the analysis of some much targeted predictors have been delineated in the current study. 

The findings of this study are in tandem with a similar study that reported a higher incidence of self-reported oral mucosal lesions in smokers residing in areas of high environmental pollution establishing a correlation between environmental CO, exhaled CO, subject’s smoking status and oral pathology incidence (Gregorczyk-maga et al., 2018). In an attempt to overcome limitations of the prior study, current study clinically assessed the presence of oral mucosal lesions by two calibrated experts and in cases requiring further confirmation available histo-pathological diagnosis was used. Other factors contributing to increased exhaled CO levels were also taken into consideration. The CO levels (15.23±7.5 ppm) were found to be significantly greater in those with an oral lesion (p=0.000) with a lower and upper 95 % CI value of 1.02 and 1.18 respectively. The smoking habits as well as cooking habits (gas stove, coal, combination of gas and kerosene) significantly predicted the exhaled CO levels (p =0.000) (Gregorczyk-maga et al., 2018).

Previous studies have correlated oral mucosal lesion incidence with smoking status wherein the smoking status was frequently confirmed by exhaled CO level (Frei et al., 2012; Moga et al., 2017). Likewise, in this study a greater percentage (67.9%) of individuals with both active and passive smoking status were diagnosed with some oral mucosal lesions. Several of the oral mucosal lesions associated with smoking have a potential risk of developing cancer. The initiation and progression of these oral lesions is dependent on the type of tobacco and the frequency and duration of its use as stated in several studies (Aishwarya et al., 2017; Aruna et al., 2011; Aslesh et.,2015). In the present study, there was a significant association between frequency of smoking and the occurrence of lesion with greater pathology observed in habitués smoking an average of 10 units/ day. This could possibly be attributed to the long-term contact of tobacco with the oral mucosa leading to prolonged exposure to the carcinogen or as a result of failure of the protective mechanism of the oral cavity.

On further questioning about the type of smoked tobacco used by the subjects in the current study it was found that majority (75.5%) who developed lesions smoked cigarettes while 18.9% them were Bidi users and rest being users of both types. A prior study conducted by Patil et al (2013) reported more than 90% prevalence of oral mucosal lesions in both Bidi and cigarette smokers. In contrast to the study by Singh et al (2010) mean CO levels here were found to be significantly higher in bidi and cigarette and bidi combined smokers in this study. 

Nicotine dependence has been reported in previous literature as a strong factor for several major causes of mortality from tobacco-related diseases. In the present study, moderate nicotine dependence significantly predicted presence of oral mucosal lesions similar to a study which had found analogous dependency amongst majority of its participants with lesions (Prashaanthi and Dharman, 2020). This could be attributed to various factors such as normalization of smoking with its initialization at an early age, lack of awareness about its harm, its glamorization by the tobacco industry which relates it with wealth and success and poorer access to tobacco cessation leading to increased difficulty in quitting (Lawson, 2003; Ekpu and Brown, 2015). Moreover, in the present study exhaled CO levels were greatest amongst smokers highly dependent on nicotine which is in line with a previous study wherein this correlation (r=0.374) was found to be significant (Sugavanesh and Pushpanjali, 2018).

BMI measures healthy weight on the basis of the height of an individual. For most adults, a BMI greater than 24.9 is considered as overweight. BMI, physical activity and co-morbidities are closely related to one another. Higher BMI has been associated with less physical activity in several studies.(Bann et al., 2020) Prior studies have also established that higher incidence of co-morbidities is seen in obese and overweight individuals (Guh et al., 2009). It has been stated in a study conducted by Zavorsky et al., (2012) that physical activity and hyperventilation increases CO clearance from the blood. The association between exhaled CO levels, co morbidities and BMI has been established in a study by Gregorczyk-Maga (2018).

During the course of conduction of the study, certain shortcomings were identified which could be taken into consideration while conducting similar studies in the future. An inherent limitation of the study was that the method of cooking, physical activities, frequency, duration and history of tobacco use etc. was self-reported by the participants which may have been biased. Further, we have not taken into consideration the environmental levels of CO which has a potential to affect the exhaled CO levels in these individuals. In the present study, 98.84% of the recruited smokers were males creating a huge gap in gender ratio. Thus, further studies are recommended with a larger sample size on different population groups.

Similarly, studies can be conducted among individuals using smokeless forms of tobacco wherein cotinine tests which use body fluids like saliva, urine and plasma can be used to replace exhaled CO monitoring to determine tobacco use. 

In conclusion, this study has established a robust correlation between exhaled CO levels and incidence of oral mucosal lesions. It is important to note the multifactorial tenacity of exhaled CO levels, which is not solely affected by smoked form of tobacco but also critical variables such as physical activity, co-morbidities, BMI, cooking habits and type of smoking habit. 

## Author Contribution Statement

1. Conception or design of the work – Dr. Nidhi Naik, Dr. Ridhima Gaunkar, Dr. Amita Kenkre Kamat. 2. Data collection - Dr. Nidhi Naik, Dr. Ridhima Gaunkar, Dr. Vinayak kamath, Dr. Akshatha Gadiyar. 3. Data analysis and interpretation – Dr. Nidhi Naik , Dr. Ridhima Gaunkar , Dr. Prachi Mungi. 4. Drafting the article - Dr. Nidhi Naik , Dr. Ridhima Gaunkar, Dr. Vinayak kamath, Dr. Akshatha Gadiyar, Dr. Prachi Mungi. 5. Critical revision of the article – Dr.Amita Kenkre Kamat.
